# mGlu2 metabotropic glutamate receptors restrain inflammatory pain and mediate the analgesic activity of dual mGlu2/mGlu3 receptor agonists

**DOI:** 10.1186/1744-8069-7-6

**Published:** 2011-01-14

**Authors:** Magda Zammataro, Santina Chiechio, Michael C Montana, Anna Traficante, Agata Copani, Ferdinando Nicoletti, Robert W Gereau

**Affiliations:** 1 Department of Drug Sciences, University of Catania; Italy; 2 Ph.D Program in Neuropharmacology, University of Catania; Italy; 3 Washington University Pain Center and Department of Anesthesiology, Washington University School of Medicine, St. Louis, Missouri, USA; 4 I.N.M. Neuromed, Pozzilli; Italy; 5 Department of Physiology and Pharmacology, University of Rome "Sapienza", Italy

## Abstract

Group II metabotropic glutamate receptors (mGluRs) couple to the inhibitory G-protein Gi. The group II mGluRs include two subtypes, mGlu2 and mGlu3, and their pharmacological activation produces analgesic effects in inflammatory and neuropathic pain states. However, the specific contribution of each one of the two subtypes has not been clarified due to the lack of selective orthosteric ligands that can discriminate between mGlu2 and mGlu3 subtypes.

In this study we used mGlu2 or mGlu3 knock-out mice to dissect the specific role for these two receptors in the endogenous control of inflammatory pain and their specific contribution to the analgesic activity of mixed mGlu2/3 receptor agonists.

Our results showed that mGlu2^-/- ^mice display a significantly greater pain response compared to their wild type littermates. Interestingly the increased pain sensitivity in mGlu2^-/- ^mice occurred only in the second phase of the formalin test. No differences were observed in the first phase. In contrast, mGlu3^-/- ^mice did not significantly differ from their wild type littermates in either phase of the formalin test.

When systemically injected, a single administration of the mGlu2/3 agonist, LY379268 (3 mg/kg, ip), showed a significant reduction of both phases in wild-type mice and in mGlu3^-/- ^but not in mGlu2^-/- ^mice. However tolerance to the analgesic effect of LY379268 (3 mg/kg, ip) in mGlu3^-/- ^mice developed following 5 consecutive days of injection.

Taken together, these results demonstrate that: (i) mGlu2 receptors play a predominant role over mGlu3 receptors in the control of inflammatory pain in mice; (ii) the analgesic activity of mixed mGlu2/3 agonists is entirely mediated by the activation of the mGlu2 subtype and (iii) the development of tolerance to the analgesic effect of mGlu2/3 agonists develops despite the lack of mGlu3 receptors.

## Finding

Metabotropic glutamate (mGlu) receptors are considered promising targets in the treatment of chronic pain. All mGlu receptor subtypes (mGlu1-8), except mGlu6, are widely distributed along the pain neuraxis, and modulate cellular mechanisms of nociceptive sensitization that underlie the development of chronic pain [1-3]. We and others have focused on the role of group-II mGlu receptors (mGlu2 and mGlu3), which are coupled to Gi proteins and depress pain transmission at synapses between primary afferent fibers and second order sensory neurons in the dorsal horn of the spinal cord [4,5]. mGlu2/3 receptors are also found in peripheral nociceptors, where their activation reduces hyperalgesia by limiting cAMP-dependent regulation of TRPV1 channels and TTX-resistant sodium channels [6-9]. The analgesic role of mGlu2/3 receptors is supported by *in vivo *studies with LY354740, LY379268, and LY389795, which activate both receptor subtypes with high potency and are systemically active [10]. Systemic treatment with these drugs causes analgesia in models of inflammatory and neuropathic pain [11-13]. While activation of group II mGlu receptors has been shown to be analgesic, the individual role of mGlu2 and mGlu3 receptors in the regulation of pain threshold has not been dissected, as yet. This issue is relevant from a therapeutical standpoint due to the increasing availability of allosteric ligands that discriminate between the two receptor subtypes [14].

**Figure 1:**
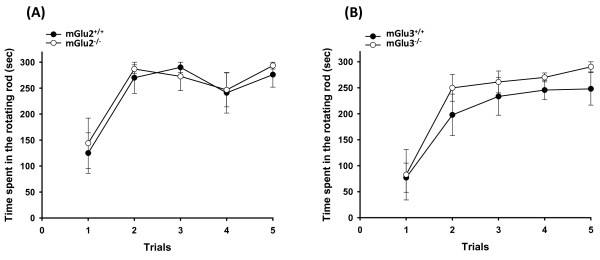
**Motor performance of mGlu2^-/- ^and mGlu3^-/- ^mice on the accelerating rotarod**. **(A) **mGlu2^-/- ^mice and **(B) **mGlu3^-/- ^mice were tested along with wild type littermates. Motor performance was tested by using the accelerating rotarod in five consecutive trials with 15 min inter-trial interval. Results indicate the time (seconds) that mice remained on the rotating rod before falling. No differences were observed between mGlu2^-/- ^mice or mGlu3^-/- ^mice and their respective wild-type littermates. p > 0.7 Two-way ANOVA followed by Fisher's *post hoc *analysis.

In this study we used adult (7-8 weeks) male mGlu2^-/- ^and mGlu3^-/- ^mice on a C57BL/6J background. mGlu2^-/- ^mice were generated in the lab of Prof. Shigetada Nakanishi at Kyoto University, Japan. mGlu3^-/- ^mice were purchased from Jackson Laboratories. Knockout mice were backcrossed with C57BL/6J wildtype mice for the generation of mGlu2^+/- ^and mGlu3^+/- ^mice. Knockout mice and their wildtype littermates generated by heterozygous crosses were used in our experiments. Mice were housed 5 animals per cage with food and water *ad libitum *in standard 12/12 h light/dark cycle, and were manipulated daily for an adaptation period of 2 weeks before testing. (1*R*,4*R*,5*S*,6*R*)-4-Amino-2-oxabicyclo[3.1.0]hexane-4,6-dicarboxylic acid (LY379268; purchased from Tocris Cookson) was dissolved in saline. Different groups of mGlu2^-/- ^mice, mGlu3^-/- ^mice, and their wild-type littermates (n = 8-12) were treated as follows: (i) acute injection of LY379268 (3 mg/kg, i.p.) or saline 30 min prior to behavioral testing; (ii) repeated injections of LY379268 (3 mg/kg, i.p. once daily for 5 days) or saline (only in mGlu3^-/- ^mice and their wild-type littermates), with mice being tested 30 min after the last injection. All experiments were carried out according to the recommendations of Institutional Animal Care and Use Committee (IACUC). All efforts were made to minimize animal suffering and to reduce the number of animals used. Motor performance was assessed on an accelerating rotarod treadmill (Ugo Basile, Comerio, Italy) as described previously [15]. mGlu2^-/- ^and mGlu3^-/- ^mice did not show differences in motor performance with respect to their wild-type littermates on the rotarod test (Figure 1).

Inflammatory pain was assessed using the formalin test. A 2% formalin solution (10 μl) was injected s.c. into the plantar surface of the right hind paw. After the injection, mice were immediately placed in a plexiglas box (20 × 15 × 15 cm) surrounded by mirrors to allow the observation of nociceptive responses that include licking, lifting and shaking of the injected paw. Tests were performed between 08:00 h and 12:00 h to minimize variability. After formalin injection, mice were observed for 1 h and their behaviors were recorded by researchers blind to genotypes and drug treatments. Formalin scores were separated into two phases, phase I (0-10 min) and phase II (15-50 min). The mean behavioral score was calculated in blocks of 5 min for each of the two phases. A mean response was then calculated for each phase.

**Figure 2:**
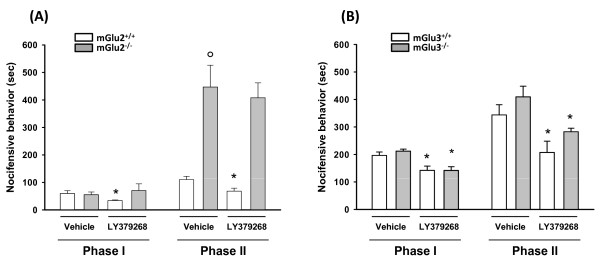
**The formalin test in mGlu2^-/- ^and mGlu3^-/- ^mice**. **A**, Enhanced pain behavior in mGlu2^-/- ^mice after formalin injection. The first phase (0 - 10 min) of pain behavior (defined as duration of licking responses) did not differ between mGlu2^+/+ ^mice and mGlu2^-/- ^mice after formalin injection, but the second phase (10 - 50 min) was significantly enhanced in mGlu2^-/- ^mice. The acute injection of LY379268 (3 mg/kg i.p.) 30 minutes before formalin injection significantly reduced both phases in mGlu2^+/+ ^mice but not in mGlu2^-/- ^mice. **B**, mGlu3^-/- ^mice did not significantly differ from littermate mGlu3^+/+ ^mice in either of the two phases of the formalin test. The acute injection of LY379268 (3 mg/kg i.p.) 30 minutes before formalin injection elicited a significant reduction of both phases in mGlu3^+/+ ^mice and in mGlu3^-/- ^mice. Data represent the mean ± S.E.M. of 8 to 12 mice per group. *p < 0.05 versus the respective vehicle group, ° *p *< 0.001 vs wild-type mice (two-way ANOVA)

**Figure 3:**
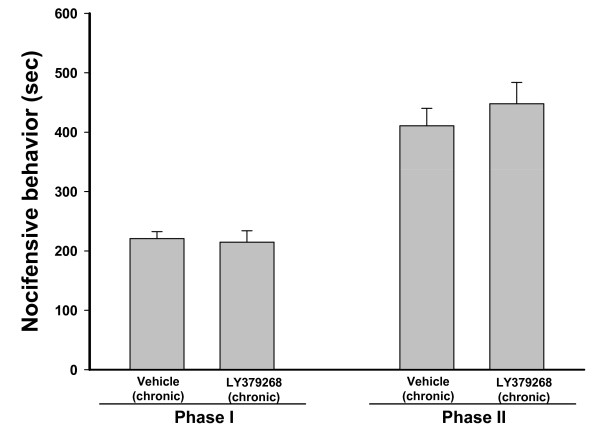
**Loss of the analgesic effect of LY379268 in mGlu3^-/- ^mice following repeated dosing**. Vehicle or LY379268 (3 mg/kg, i.p.) were injected for 4 days every 24 hours in mGlu3^-/- ^mice. On day 5 vehicle or LY379268 were administered 30 min before formalin injection. Data represent the mean ± S.E.M. of 8 to 12 mice. *p *> 0.3 vs vehicle group (*t*-test).

We found no difference in the first phase of the formalin test between mGlu2^-/- ^and wild-type mice. In contrast, mGlu2^-/- ^mice showed a 4-fold increase in the second phase of the formalin test as compared to wild-type littermates (Figure 2A). Acute injection of LY379268 in wild-type mice caused analgesia in both phases of the formalin test, as expected [16]. Remarkably, the drug was totally ineffective in mGlu2^-/- ^mice (Figure 2A). In contrast to mice lacking mGlu2 receptors, mGlu3^-/- ^mice did not show significant differences in the first or second phase of the formalin test as compared to their wild-type littermates (Figure 2B). In mGlu3^-/- ^mice acute treatment with LY379268 caused analgesia both in the first and second phases of the formalin test similar to that seen in wild-type mice (Figure 2B). Previous studies have shown that tolerance to LY379268 develops following repeated dosing in rats [13]. Thus we also examined whether tolerance to the analgesic activity of LY379268 could be affected by a lack of mGlu3 receptors (mGlu2^-/- ^mice were not tested because they did not respond to LY379268). A 5-day treatment with LY379268 failed to cause analgesia in the first or second phase of the formalin test in mGlu3^-/- ^mice (Figure 3), suggesting that tolerance to the drug developed in spite of the lack of mGlu3 receptors.

Taken together, our data indicate that endogenous activation of mGlu2 receptors negatively regulate inflammatory pain and mediate the analgesic response to dual mGlu2/3 receptor agonists. These findings are in agreement with the evidence that L-acetylcarnitine and histone deacetylase inhibitors cause analgesia by up-regulating the expression of mGlu2 receptors in the dorsal root ganglia and spinal cord, without affecting the expression of mGlu3 receptors [16-18]. A role for mGlu3 receptors in pain has been suggested by studies showing an increased expression of mGlu3 receptor protein in the cerebral cortex and spinal cord in response to peripheral inflammation [19-21], and by the analgesic activity of specific inhibitors of N-acetylated-α-linked acidic dipeptidase (NAALADase), the enzyme that degrades the putative mGlu3 receptor agonist, N-acetylaspartylglutamate (NAAG) [22-28]. However, the activity of NAAG at mGlu3 receptors has recently been questioned [29].

The mechanisms underlying the selective involvement of mGlu2 (vs. mGlu3) receptors in pain control remain to be addressed. mGlu2 receptors are uniquely localized in neurons and particularly in the pre-terminal region of axons, where they negatively modulate neurotransmitter release; in contrast, mGlu3 receptors are found in both presynaptic and postsynaptic sites as well as in glial cells [30]. Activation of glial mGlu3 receptors might stimulate the production of neurotrophic factors, such as brain-derived neurotrophic factor (BDNF) and nerve growth factor (NGF) [31,32] that cause hyperalgesia and may therefore counterbalance a potential analgesic effect of mGlu3 receptors [33]. This interesting hypothesis warrants further investigation.

In conclusion we propose that: (i) mGlu2 receptors play a predominant role over mGlu3 receptors in the endogenous control of inflammatory pain and (ii) the analgesic effects observed with the group II mGlu receptor agonist LY379268 in the formalin test are mediated by activity at mGlu2 and not mGlu3.

Our results are promising for a potential use of selective activators of mGlu2 receptors (e.g., mGlu2 receptor enhancers) in pain treatment, providing that these drugs show a favorable profile of tolerability in humans. mGlu2^-/- ^mice show an impairment of hippocampal mossy fibre long-term depression (LTD), although they perform normally in water maze learning tasks [34]. Experiments combining the mGlu2/3 receptor agonist, LY354740, and mGlu2^-/- ^mice show that activation of mGlu2 receptors causes some cognitive impairment (i.e. a deficit in delayed matching and non-matching to position) and impairment in spatial learning [35]. However, a 6-day treatment with the mGlu2/3 receptor *antagonist*, (2S)-α-ethylglutamate, results in an impairment of long-term (reference) memory in the eight-arm radial maze [36], whereas no deficits in working memory are seen in humans treated with LY354740 [37]. Therefore, it is not entirely clear what side effect profile might be anticipated for mGlu2 modulators. Therefore, the potential utility of mGlu2 receptor enhancers in the treatment of chronic pain will be informed by data from clinical trials with these drugs in the treatment of schizophrenia and other disorders.

## Competing interests

The authors declare that they have no competing interests.

## Authors' contributions

The study was conceived and the experiments were designed by SC, MZ, AC, FN, and RWG. MZ, SC and MCM performed behavioral experiments and analysis. AT performed genotyping for the mGlu2^-/- ^colony. MZ, SC, and MCM made the figures. All authors contributed to writing the manuscript, and all read and approved the final manuscript.
